# Cardiac Hypertrophy in Pregnant Rats, Descendants of Fructose-Fed Mothers, an Effect That Worsens with Fructose Supplementation

**DOI:** 10.3390/foods13182944

**Published:** 2024-09-18

**Authors:** Cristina Donis, Elena Fauste, Madelín Pérez-Armas, Paola Otero, María I. Panadero, Carlos Bocos

**Affiliations:** Facultad de Farmacia, Universidad San Pablo-CEU, CEU Universities, Montepríncipe, Boadilla del Monte, 28668 Madrid, Spain; c.donis@usp.ceu.es (C.D.); elena.faustealonso@ceu.es (E.F.); m.perez170@usp.ceu.es (M.P.-A.); paotero@ceu.es (P.O.); ipanade@ceu.es (M.I.P.)

**Keywords:** fructose, pregnancy, cardiac hypertrophy, fetal programming

## Abstract

The role of fructose consumption in the development of obesity, MetS, and CVD epidemic has been widely documented. Notably, among other effects, fructose consumption has been demonstrated to induce cardiac hypertrophy. Moreover, fructose intake during pregnancy can cause hypertrophy of the maternal heart. Our previous research has demonstrated that maternal fructose intake has detrimental effects on fetuses, which persist into adulthood and are exacerbated upon re-exposure to fructose. Additionally, we found that maternal fructose consumption produces changes in female progeny that alter their own pregnancy. Despite these findings, fructose intake during pregnancy is not currently discouraged. Given that cardiac hypertrophy is a prognostic marker for heart disease and heart failure, this study aimed to determine whether metabolic changes occurring during pregnancy in the female progeny of fructose-fed mothers could provoke a hypertrophic heart. To test this hypothesis, pregnant rats from fructose-fed mothers, with (FF) and without (FC) fructose supplementation, were studied and compared to pregnant control rats (CC). Maternal hearts were analyzed. Although both FF and FC mothers exhibited heart hypertrophy compared to CC rats, cardiac DNA content was more diminished in the hearts of FF dams than in those of FC rats, suggesting a lower number of heart cells. Accordingly, changes associated with cardiac hypertrophy, such as HIF1α activation and hyperosmolality, were observed in both the FC and FF dams. However, FF dams also exhibited higher oxidative stress, lower autophagy, and decreased glutamine protection against hypertrophy than CC dams. In conclusion, maternal fructose intake induces changes in female progeny that alter their own pregnancy, leading to cardiac hypertrophy, which is further exacerbated by subsequent fructose intake.

## 1. Introduction

Fructose, a natural sugar found in fruits, vegetables, and honey, is extensively used in the food industry, especially in sugary soft drinks and industrially processed foods. In Europe, these beverages typically contain sucrose, which is a disaccharide made up of fructose linked to glucose. In contrast, the USA has increasingly adopted the use of high-fructose corn syrup (HFCS) as the main sweetener in sugary drinks. HFCS is a mixture of both free monosaccharides [1,2]. Fructose is sweeter than sucrose or glucose but has a lower glycemic index. However, recent scientific evidence has shifted the view of fructose from being neutral or beneficial to potentially harmful [1]. Nowadays, excessive consumption of added fructose is associated with lifestyle-induced health issues such as obesity, metabolic dysfunction-associated fatty liver disease (MAFLD), type 2 diabetes (T2D), cardiovascular disease (CVD), and hyperuricemia [3].

In recent decades, global fructose consumption has significantly increased. This trend parallels a notable increase in the prevalence of cardiovascular conditions, including cardiac hypertrophy, high blood pressure, and insulin resistance [4]. Cardiac hypertrophy is a term that broadly describes the enlargement of the myocardium in response to a sustained increase in blood pressure or blood volume [5]. It can be classified into two types: physiological and pathological. Both forms of hypertrophy initially emerge as adaptive reactions to cardiac stress. While physiological hypertrophy preserves cardiac function over the long term, pathological hypertrophy is associated with adverse cardiovascular outcomes, such as heart failure, arrhythmias, and increased mortality [6].

Since fructose is primarily metabolized by the liver, research on its potential contribution to heart disease is limited [7]. However, recent studies have revealed potential links between dietary factors and heart disease progression. Indeed, it has been suggested that oxidative stress may serve as a connecting factor between high carbohydrate intake and heart disease development [4]. Moreover, excessive consumption of dietary fructose has been associated with the early onset of diastolic dysfunction, changes in the expression of mitochondrial proteins, apoptosis, and the accumulation of lipid compounds in the heart [8]. Both clinical and animal studies have demonstrated that excessive fructose consumption is linked to the development of diabetes, cardiovascular disease, and metabolic syndrome [8].

Fructose metabolism can produce sorbitol, which is a well-known osmolyte. Fructolysis also yields trioses-phosphate, which can be converted into lipids [9]. In addition, fructose is a recognized source of reactive oxygen species that promotes oxidative stress [10]. Interestingly, changes in osmolality, steatosis, and oxidative stress have been related to hypertrophic situations [11]. Moreover, it has been recently described that cardiac pathologic stress increases hypoxia-inducible factor (HIF1α) and its target genes. Curiously, HIF1α also regulates fructose metabolism, and it is known that fructolysis supports anabolic growth that promotes cardiac hypertrophy [12]. Annandale et al. (2021) have recently published a review where they discuss several mechanisms through which fructose consumption can promote cardiac hypertrophy [8]. Among others, fructose has been shown to provide a fuel source for the stressed heart. Pathological stress leads to increased expression of HIF1α and activation of HIF1α-target genes encoding both glycolytic enzymes and specific splicing factors, which in turn activate local fructose metabolism, fueling nucleic acid, lipid, and protein biosynthetic pathways that are essential for hypertrophic growth, steatosis, and cardiac dysfunction [7,8]. Furthermore, elevated cardiac sorbitol content has been related to the progression of diabetic cardiomyopathy. Indeed, cardiac contractile dysfunction and oxidative stress under high glucose conditions are ameliorated through the inhibition of the polyol pathway, which converts glucose into sorbitol and/or fructose [8]. Moreover, elevated circulating fructose levels and intracellular fructose production can induce metabolic disturbances and oxidative stress in cardiac mitochondria. According to this, the treatment with antioxidants, such as tempol, resveratrol, polyphenolic compounds, and SkQ1, which protect mitochondria from oxidative damage, has been proven to alleviate cardiac hypertrophy [4,8]. Most experiments linking fructose and cardiac tissue hypertrophy [11] have been carried out in non-pregnant animals. However, Olaniyi and Olatunji (2019) [13] observed cardiac hypertrophy in fructose-fed pregnant rats. They found glucose dysregulation, oxidative stress, and increases in pyruvate dehydrogenase kinase-4 (PDK4), along with the accumulation of lipids, glycogen, and lactate in the heart. All these fructose-induced effects were linked to cardiac hypertrophy. 

During the initial phases of growth and development, environmental and nutritional stressors can induce changes in tissue structure and function that persist into adulthood through a phenomenon known as fetal programming [14]. Recent research has revealed that increased dietary fructose consumption during pregnancy can trigger a fetal programming process that occurs through epigenetic modifications (such as cytosine methylation of DNA and microRNAs) that take place early in life without affecting the DNA sequence [15,16]. This phenomenon has been shown to contribute to a range of adverse outcomes in the offspring, including hypertension, obesity, diabetes, CVD, and MAFLD [15,17].

Previous studies have shown that adverse conditions experienced during fetal development, such as hypoxia, can lead to lasting disruptions in heart function [18]. Additionally, several investigations have suggested that adverse maternal nutritional status during pregnancy, either due to undernutrition or overnutrition, can result in persistent effects on heart health, including ventricular hypertrophy and changes in gene expression patterns in the fetal left ventricle, leading to cardiac hypertrophy and fibrosis [14]. In summary, alterations in metabolic conditions during pregnancy can have adverse consequences on cardiac health through fetal cardiac programming that could potentially persist into adulthood.

However, the effect of maternal fructose intake on the heart health of offspring has hardly been studied. To the best of our knowledge, only one report [14] has demonstrated that cardiac remodeling and myocardial hypertrophy are exacerbated after ventricular pressure overload in males from fructose-fed mothers, in comparison to males from control mothers. Nevertheless, this report has only investigated the effect on the male offspring of mothers who were fed solid fructose during pregnancy and lactation, and the descendants were also subjected to conditions that provoked heart hypertrophy. Therefore, it has not yet been reported whether this cardiac hypertrophy also occurs in female offspring when they become pregnant as a condition that also causes heart hypertrophy. 

Given that high fructose intake is not contraindicated during the maternal period, the aims of the present study are (1) to study whether maternal fructose intake affects cardiac hypertrophy in female offspring during pregnancy and (2) to determine whether fructose consumption during pregnancy can increase the risk of heart hypertrophy in descendants of fructose-fed dams.

## 2. Materials and Methods

### 2.1. Animals and Experimental Design

An animal model of maternal liquid fructose intake was used as previously described [19,20]. The experimental protocols were approved by the Animal Research Committee of the University San Pablo-CEU, Madrid, Spain (ref. numbers 10/206458.9/13 and 10/042445.9/19). Pregnant rats were randomly divided into a control group (with no supplementary sugar in the drinking water) and a fructose-fed group (receiving fructose at 10% wt/vol in the drinking water) throughout gestation (five rats per group). Both groups were fed a standard rat chow diet (B&K Universal; Barcelona, Spain). After delivery, female pups continued to be fed a standard chow diet and tap water with no additives. At 2 months of age, female progeny from the control and fructose-fed mothers were mated with control males. Pregnant rats from control mothers were kept in tap water with no supplementary sugar during their gestation and were considered the CC group. On the other hand, pregnant rats from fructose-fed mothers were randomly separated into two groups: one group received water with no additives (FC), and another group drank 10% fructose (wt/vol) in water (FF) throughout gestation (five rats per group). To reduce the “litter effect”, animals within each experimental group were born to different dams. Regarding the nomenclature of the different experimental groups, the first letter indicates whether the mothers (F0 generation) had been supplied with tap water during pregnancy (C, control) or water containing fructose (F, fructose), and the second letter indicates whether the progeny (F1 generation) received fructose (F) or not (C) during their own pregnancy.

Pregnant rats were euthanized on the 21st day of gestation at 10:00 h. Before sacrifice, food and liquid fructose was removed at 8:00 h. Maternal livers, kidneys, and hearts were obtained, weighed, and frozen.

### 2.2. DNA Extraction and Quantification

For the extraction of total deoxyribonucleic acid (DNA) present in the heart, between 15 and 20 mg of heart tissue and the DNeasy Blood and Tissue Kit (Qiagen, Germantown, MD, USA) were used following the manufacturer’s instructions. DNA purity and integrity were determined using NanoDrop One (ThermoFisher, Waltham, MA, USA) and gel electrophoresis, respectively. DNA concentrations were quantified by NanoDrop One (ThermoFisher, Waltham, MA, USA) and corrected by tissue weight.

### 2.3. Lipid Extraction and Triglycerides Determination

Two hundred milligrams of heart were immersed in chloroform: methanol 2:1 plus dibutylhydroxytoluene (BHT) and used for lipid extraction following the Folch method [21]. Aliquots of lipid extracts were dried, and the remaining residue was redissolved in isopropanol and used to determine cholesterol using a Cholesterol CHOD-POD kit (Spinreact, Girona, Spain).

Next, the triglyceride concentration was determined following the method proposed by Carr et al. (1993) [22] with some modifications. Six hundred microliters of the lipid extract were mixed with 1.25% Triton X-100 solution in chloroform. Subsequently, the solvent was removed by evaporation using a SpeedVac (ThermoFisher, Waltham, MA, USA) until it was completely dry. Then, the samples were reconstituted in distilled water and incubated at 37 °C for 15 min. Triglycerides were measured using a Triglycerides GPO-POD kit (Spinreact, Girona, Spain).

### 2.4. Lactate and Uric Acid Quantification

One hundred milligrams of the heart were homogenized in 1.2 mL of PBS using a TissueLyser LT (Qiagen, Germantown, MD, USA). After centrifugation, the supernatant was collected, deproteinized using 20% trichloroacetic acid (TCA), and neutralized with 2% NaOH. Next, lactate and uric acid concentrations were determined in deproteinized PBS homogenates using Lactate LO-POD and Uric Acid-LQ Uricase-POD kits, respectively (Spinreact, Girona, Spain).

### 2.5. Protein Carbonyls Determination

To determine protein carbonyls, a marker of protein oxidation, the method described by Levine et al. (1990) [23] was used, based on the reaction of 2,4-dinitrophenylhydrazine (DNPH) with carbonyl groups, forming dinitrophenylhydrazone adducts that can be detected at 340 nm. Briefly, heart tissue was homogenated in PBS, and supernatants (0.5 mg protein) were incubated with 10 mM DNPH in 2 M HCl for 1h at room temperature and protected from light. In parallel, blanks were prepared by incubating each sample without 10 mM DNPH, but with 2 M HCl. Afterward, proteins were precipitated with 20% tricloroacetic acid (TCA) and isolated by cold centrifugation. The precipitates were washed three times with 1:1 ethanol:ethyl acetate and resuspended in 6 M guanidine pH 2.5. Dinitrophenylhydrazone adducts were measured at 340 nm using a SpectroStar Nano (BMG Labtech, Ortenberg, Germany). Additionally, the data were corrected by the number of proteins present in the samples using the colorimetric Protein in Urine and CSF kit (Spinreact, Girona, Spain).

### 2.6. RNA Extraction and Gene Expression Determination by qPCR

Total RNA was isolated from the heart using Ribopure (Invitrogen, ThermoFisher Scientific, Waltham, MA, USA). Total RNA was subjected to DNase I treatment using Turbo DNA-free (Invitrogen, ThermoFisher Scientific, Waltham, MA, USA), and RNA integrity was confirmed by agarose gel electrophoresis. Afterward, cDNA was synthesized by oligo(dT)-primed reverse transcription with Superscript II (Invitrogen, ThermoFisher Scientific, Waltham, MA, USA). Briefly, 250 ng RNA, oligo(dT)_12-18_ (Invitrogen, ThermoFisher Scientific, Waltham, MA, USA), and dNTP mix were incubated at 65 °C for 5 min. After that, 5× First-Strand Buffer and 0.1 M DTT were added and incubated at 42 °C for 2 min. Finally, cDNA was synthesized by adding SuperScriptTM II RT followed by incubation at 42 °C for 50 min and a further inactivation at 70 °C for 15 min. qPCRs were performed using a CXF96 Touch (Bio-Rad, Hercules, CA, USA). The reaction solution was carried out in a volume of 20 μL, containing 10 pmol of both forward and reverse primers, 10× SYBR Premix Ex Taq (Takara Bio Inc., Kusatsu, Shiga, Japan), and the appropriate nanograms of the cDNA stock. The protocol for cDNA amplification by qPCR was as follows: one cycle at 95 °C for 3 min, 40 cycles at 95 °C for 10 s, and 60 °C for 30 s. Rps29 was used as the reference gene for qPCR. Primer sequences were obtained either from the Atlas RT-PCR Primer Sequences (Clontech, Palo Alto, CA, USA) or designed using Primer Blast (NCBI, Bethesda, MD, USA). Samples were analyzed in duplicate for each assay. Amplification of non-specific targets was discarded using the melting curve analysis method for each amplicon. qPCR efficiency and linearity were assessed by optimization of the standard curves for each target. The transcription was quantified with CFX Maestro 2.0 software (Bio-Rad, Hercules, CA, USA) using the efficiency correction method [24].

### 2.7. Statistical Analysis

Results are expressed as the mean ± S.E. Treatment effects were analyzed by one-way analysis of variance (ANOVA). When treatment effects were significantly different (*p* < 0.05), means were tested by Tukey’s multiple range test using the computer program SPSS, version 28. When the variance was not homogeneous, a post-hoc Tamhane test was performed. Significant differences between the experimental groups are indicated using different letters.

## 3. Results

### 3.1. Descendants of Fructose-Fed Mothers Subjected or Not to Liquid Fructose Showed Maternal Cardiac Hypertrophy

In a previous report [19], we observed that pregnancy in progeny from fructose-fed mothers (FC) and ingestion of liquid fructose throughout gestation in pregnant rats from fructose-fed mothers (FF) diminished insulin sensitivity and provoked hyperleptinemia and lipid accretion in both the liver and placenta. Interestingly, Olaniyi and Olatunji (2019) [13] found that liquid fructose exposure during gestation induced disrupted glucose homeostasis, oxidative stress, and increased lipid, glycogen, uric acid, and lactate cardiac contents, and all of these changes were associated with cardiac hypertrophy.

Accordingly, the current report is a follow-up study to investigate the effects of pregnancy in descendants of fructose-fed mothers subjected (FF) or not (FC) to fructose exposure on maternal cardiac hypertrophy and related parameters.

Although both maternal body weight and conceptus weight at the end of gestation remained unchanged between the three groups [19], maternal body weight (conceptus free) turned out to be lower in descendants from fructose-fed mothers, becoming significant for the FC dams versus the CC group (Table 1). In accordance with this, maternal liver weight tended to be lower and maternal kidney weight was unchanged in pregnant rats from fructose-fed dams compared to the control group (CC) (Table 1). Curiously, the weight of maternal hearts showed the opposite profile, since it tended to increase in both FC and FF mothers versus CC (Table 1). This increase observed in FC was of the same magnitude as that found by Olaniyi and Olatunji (2019) [13] in fructose-fed dams. However, it is important to emphasize that FC dams were not exposed to fructose during their own pregnancy. As expected, the increase found in the FF group was even higher than that observed in the FC group (Table 1), and this could mean that the effect of maternal fructose intake was exacerbated by re-exposure to sugar.

**Figure 1:**
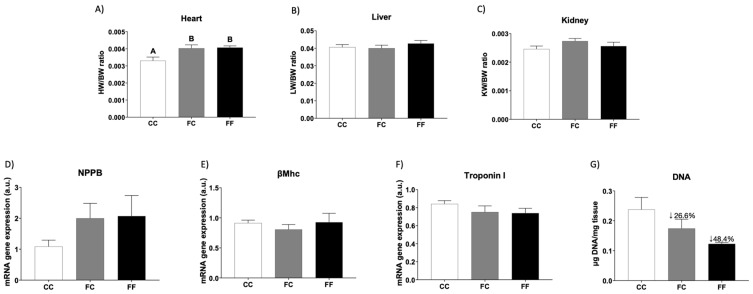
Pregnancy in progeny from fructose-fed mothers and ingestion of liquid fructose throughout gestation in pregnant rats from fructose-fed mothers induce cardiac hypertrophy. (**A**) Heart weight corrected by BW, (**B**) liver weight corrected by BW, and (**C**) kidney weight corrected by BW of pregnant rats from control mothers (CC, empty bar) or pregnant rats from fructose-fed mothers subjected (FF, black bar) or not (FC, gray bar) to fructose intake throughout own pregnancy. Heart levels of specific mRNA for hypertrophy markers: (**D**) NPPB, (**E**) βMhC, and (**F**) Troponin I gene expression. (**G**) DNA content of the tissue. The fold decrease is indicated in percentages in comparison to the CC group. Relative target gene mRNA levels were measured by Real-Time PCR as explained in Materials and Methods, normalized to Rps29 levels, and expressed in arbitrary units (a.u.). Data are expressed as the mean ± S.E., n = 5 rats. Values not sharing a common letter are significantly different (*p* < 0.05). NPPB: Natriuretic peptide B; βMhC: β Myosin heavy chain; BW: Body weight (conceptus free).

Accordingly, the ratio between heart weight and body weight (without conceptus) was significantly augmented in descendants of fructose-fed mothers, regardless of the diet consumed during their own pregnancy, when compared to the CC dams (Figure 1A). Interestingly, this finding was exclusively found in the maternal heart because the ratio of liver and kidney weights remained unaltered between the three experimental groups (Figure 1B,C). This potential cardiac hypertrophy induced by pregnancy in descendants of fructose-fed mothers and by fructose consumption during their own pregnancy coincided with an increase in the gene expression of Natriuretic peptide B (NPPB) (Figure 1D), a typical marker of cardiac hypertrophic [12]. This effect was not observed for other markers like β-Myosin heavy chain (βMhc) and troponin I (Figure 1E,F) [25]. Although the NPPB gene expression values in FC and FF duplicated those found in CC dams, these increases were not statistically significant, likely due to the high standard error observed in these two groups. Interestingly, when the DNA content of the heart was evaluated [26] as a measure of the total number of cells, a clear diminution was observed in the FC and FF groups in comparison to CC mothers (Figure 1G). Despite not being significant, the percentages of reduction observed in FC (26.6%) and FF (48.4%) versus CC would suggest that the increase in heart weight in these groups would be more related to hypertrophic cardiomyocytes rather than higher cellularity. Unfortunately, there were no tissue sections available to corroborate this. Nevertheless, other possible reasons that could explain the increase found in cardiac weight could be the accumulation of glycogen and/or lipids, as reported by Olaniyi and Olatunji (2019) [13] in gestational fructose-exposed rats.

### 3.2. Metabolic and Oxidative Stress Parameters Related to Cardiac Hypertrophy in Descendants of Fructose-Fed Mothers Subjected or Not to Liquid Fructose

Although we previously found diminished insulin sensitivity and liver steatosis [19] in progeny from fructose-fed mothers subjected (FF) or not (FC) to liquid fructose during their own pregnancy, and Olaniyi and Olatunji (2019) [13] observed that gestational exposure to liquid fructose led to insulin resistance that was accompanied by increased cardiac lipid, glycogen, uric acid, and lactate contents, we did not find any of these increases in descendants from fructose-fed dams in the present study.

**Figure 2:**
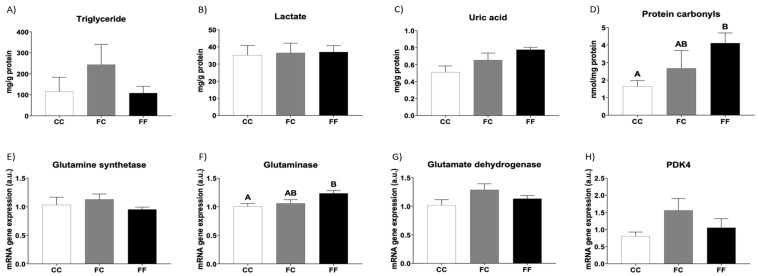
Metabolic and oxidative stress parameters related to cardiac hypertrophy in pregnant rats. Heart levels of (**A**) triglycerides, (**B**) lactate, (**C**) uric acid, and (**D**) protein carbonyls of pregnant rats from control mothers (CC, empty bar) or pregnant rats from fructose-fed mothers subjected (FF, black bar) or not (FC, gray bar) to fructose intake throughout own pregnancy. Heart levels of specific mRNA for (**E**) glutamine synthetase, (**F**) glutaminase, (**G**) glutamate dehydrogenase, and (**H**) PDK4 gene expression. Relative target gene mRNA levels were measured by Real-Time PCR as explained in Materials and Methods, normalized to Rps29 levels, and expressed in arbitrary units (a.u.). Data are expressed as mean ± S.E., n = 5 rats. Values not sharing a common letter are significantly different (*p* < 0.05). PDK4: Pyruvate dehydrogenase kinase 4.

Thus, although the content of triglycerides in the heart tended to be augmented in FC rats, it did not become significant in comparison to CC dams, and this trend was not observed in the FF group (Figure 2A). In addition, the amounts of total lipids, cholesterol, and phospholipids in the heart were similar between the three groups (Supplementary Table S1). On the other hand, the results obtained for the glycogen content turned out to be inconclusive (64.3 ± 38.3, 204.7 ± 158.1, and 210.8 ± 141.1 mg of glycogen/g of tissue, for CC, FC, and FF rats, respectively), and cardiac lactate content remained unchanged between the three experimental groups (Figure 2B). Curiously, cardiac uric acid tended to be augmented in descendants of fructose-fed mothers, although it did not reach statistical significance (Figure 2C). In line with this finding, since uric acid is a recognized prooxidant agent in tissues, protein carbonyls levels, as a marker of protein oxidative stress, were increased in progeny from fructose-fed dams, becoming significant in FF rats versus CC dams (Figure 2D).

Related to this, Olaniyi and Olatunji (2019) [13] found that all the gestational fructose-induced harmful effects observed in their study were linked to cardiac hypertrophy, and interestingly, L-glutamine prevented their apparition and also prevented cardiac hypertrophy. Therefore, we decided to measure the gene expression of enzymes related to glutamine metabolism to elucidate whether the protector agent proposed by these authors was present in the hearts of descendants from fructose-fed dams in the present study. Whereas mRNA gene expression of glutamine synthetase and glutamate dehydrogenase was not different between the three groups studied, mRNA gene expression of glutaminase, the enzyme responsible for the conversion of glutamine into glutamate and ammonium, was significantly augmented in FF dams in comparison to that in the CC group (Figure 2E–G), possibly indicating a lower amount of glutamine and, therefore, poorer protection against fructose-induced harmful effects.

In a previous study [13], the protective role of L-glutamine supplementation on gestational fructose-induced cardiac hypertrophy was related to an increase in pyruvate dehydrogenase kinase 4 (PDK4), an enzyme that regulates the entry of glycolytic products into the citric acid cycle, preventing the complete oxidation of glucose and promoting glycogen and lipid accumulation. In the present study, although PDK4 gene expression tended to be augmented in FC rats, it did not become significant in comparison to CC dams, and this effect was not observed in the FF group (Figure 2H).

### 3.3. Fructose Consumption during Pregnancy Affects the Expression of Cardiac Autophagy-Related Genes of Their Descendants

**Figure 3:**
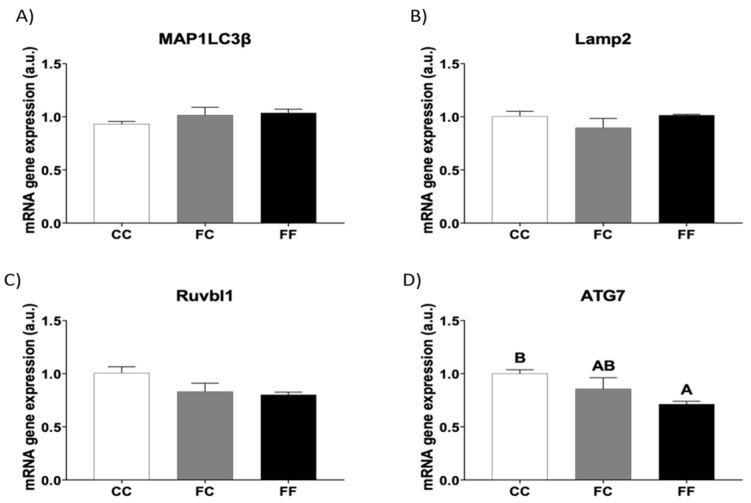
Fructose consumption during pregnancy affects the expression of autophagy-related genes in the hearts of pregnant rats from fructose-fed mothers. Heart levels of specific mRNA for: (**A**) MAP1LC3β, (**B**) Lamp2, (**C**) Ruvbl1, and (**D**) ATG7 gene expression of pregnant rats from control mothers (CC, empty bar) or pregnant rats from fructose-fed mothers subjected (FF, black bar) or not (FC, gray bar) to fructose intake throughout own pregnancy. Relative target gene mRNA levels were measured by Real-Time PCR as explained in Materials and Methods, normalized to Rps29 levels, and expressed in arbitrary units (a.u.). Data are expressed as mean ± S.E., n = 5 rats. Values not sharing a common letter are significantly different (*p* < 0.05). MAP1LC3B: Microtubule-associated protein 1 light chain 3 beta; Lamp2: Lysosomal-associated membrane protein 2; Ruvbl1: RuvB-like AAA ATPase 1; ATG7: Autophagy related 7.

Since the results previously described did not completely explain the cardiac hypertrophy found in the descendants of fructose-fed dams, we investigated whether other possible routes might be affected. Mellor et al. (2011) [27] showed in the hearts of fructose-fed mice that insulin resistance and oxidative stress could be related to upregulated autophagy and myocardial structural remodeling. However, later studies have shown controversial results, since they found that fructose feeding inhibits hepatic autophagy [28,29]. In the present study, we measured the mRNA levels of several genes related to autophagy, whose expression has been previously reported to be regulated by fructose intake [30]: microtubule-associated protein 1 light chain 3 beta (MAP1LC3b), lysosomal-associated membrane protein 2 (Lamp2), RuvB-like AAA ATPase 1 (Ruvbl1), and autophagy-related 7 (ATG7). As shown in Figure 3, whereas mRNA gene expression of MAP1LC3b and Lamp2 did not show any differences between the three experimental groups (Figure 3A,B), Ruvbl1 and ATG7 gene expression tended to be diminished in the descendants of fructose-fed dams, becoming significant for the ATG7 gene expression in FF dams in comparison to CC rats (Figure 3C,D).

### 3.4. Descendants of Fructose-Fed Mothers Subjected or Not to Liquid Fructose Present a Stimulated Cardiac Gene Expression of HIF1α Target Genes

Mirtschink et al. (2015) [12] have proposed that cardiac pathologic stress leads to an increased expression of HIF1α and HIF1α-dependent activation of genes encoding glycolytic enzymes. Moreover, fructolysis is also stimulated in this situation. Augmented fructolysis and glycolysis provide the cell with macromolecules, which are essential for hypertrophic growth, steatosis, and cardiac dysfunction [12]. Curiously, fructose metabolism generates effects similar to those induced by HIF1α, as it reduces mitochondrial function and stimulates glycolysis. Indeed, fructose has been involved in the regulation of hypoxia-inducible genes [31]. In fact, Kanbay et al. (2023) [32] have recently proposed that this interconnection between HIF1α and fructolysis pathways could produce some synergy that accelerates the development of metabolic diseases.

**Figure 4:**
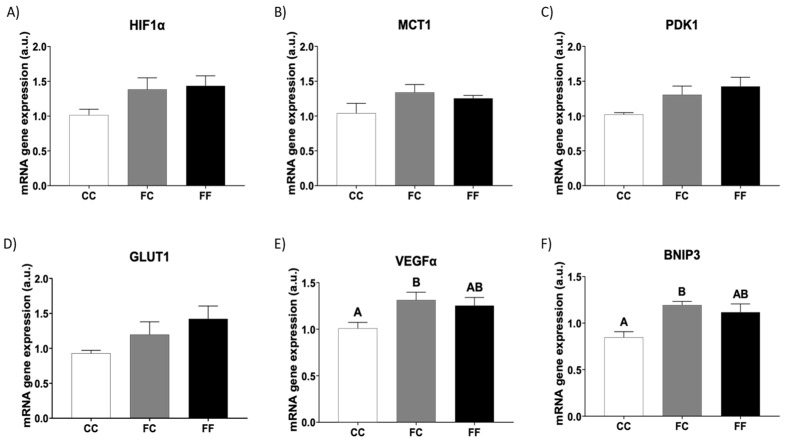
Pregnancy in progeny from fructose-fed mothers and ingestion of liquid fructose throughout gestation in pregnant rats from fructose-fed mothers stimulates cardiac gene expression of HIF1α target genes. Heart levels of specific mRNA for (**A**) HIF1α, (**B**) MCT1, (**C**) PDK1, (**D**) GLUT1, (**E**) VEGFα, and (**F**) BNIP3 gene expression of pregnant rats from control mothers (CC, empty bar) or pregnant rats from fructose-fed mothers subjected (FF, black bar) or not (FC, gray bar) to fructose intake throughout own pregnancy. Relative target gene mRNA levels were measured by Real-Time PCR as explained in Materials and Methods, normalized to Rps29 levels, and expressed in arbitrary units (a.u.). Data are expressed as mean ± S.E., n = 5 rats. Values not sharing a common letter are significantly different (*p* < 0.05). HIF1α: Hypoxia-inducible factor 1 subunit alpha; MCT1: Monocarboxylate transporter 1; PDK1: Pyruvate dehydrogenase kinase 1; GLUT1: Glucose transporter 1; VEGFA: Vascular endothelial growth factor A; BNIP3: BCL2 interacting protein 3.

However, in the present study, we did not observe that HIF1α and fructose work in synergy inducing cardiac hypertrophy. This lack of synergy could be attributed to the unchanged expression of cardiac genes encoding enzymes involved in fructose metabolism, specifically ketohexokinase, KHK, and aldolase B, ALDOB (Supplementary Table S1). Thus, although HIF1α gene expression (Figure 4A) and HIF1α target genes related to carbohydrate metabolism, such as monocarboxylate transporter 1 (MCT1), pyruvate dehydrogenase kinase 1 (PDK1), and glucose transporter 1 (GLUT1), tended to be increased in FC and FF dams, the differences were not significant compared to those in the CC group (Figure 4B–D). Other HIF1α target genes, such as lactate dehydrogenase A (LDHA) and 6-Phosphofructo-2-Kinase/Fructose-2,6-Biphosphatase 3 (PFKB3), did not show significant differences between the three experimental groups (Supplementary Table S1). Curiously, gene expression of other HIF1α target genes whose chronic activation can lead to cardiac pathologies [7,33], such as vascular endothelial growth factor alpha (VEGFα) and BCL2 interacting protein 3 (BNIP3), was augmented in descendants of fructose-fed mothers, but only becoming significant in FC dams compared to the control group (Figure 4E,F). Therefore, we have demonstrated that the addition of fructose to the diet of the progeny from fructose-fed mothers did not produce any additional effect on the increase already observed in descendants of fructose-fed dams once they became pregnant.

### 3.5. Descendants of Fructose-Fed Mothers Subjected or Not to Liquid Fructose Present Altered Expression of Cardiac Genes Related to Osmolality

**Figure 5:**
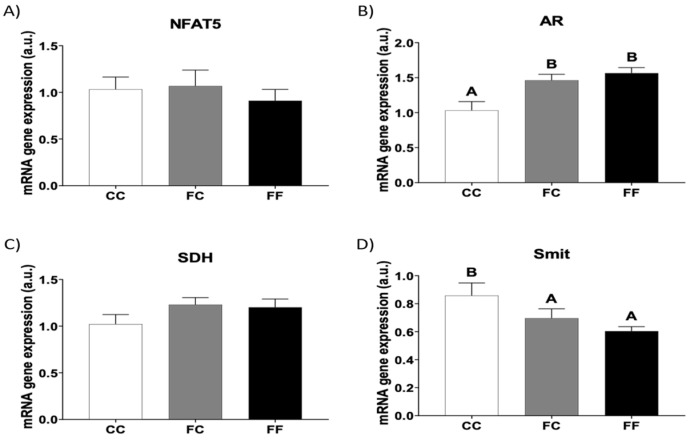
Pregnancy in progeny from fructose-fed mothers and ingestion of liquid fructose throughout gestation in pregnant rats from fructose-fed mothers influence the expression of cardiac genes related to osmolality. Heart levels of specific mRNA for (**A**) NFAT5, (**B**) AR, (**C**) SDH, and (**D**) Smit gene expression in pregnant rats from control mothers (CC, empty bar) or pregnant rats from fructose-fed mothers subjected (FF, black bar) or not (FC, gray bar) to fructose intake throughout own pregnancy. Relative target gene mRNA levels were measured by Real-Time PCR as explained in Materials and Methods, normalized to Rps29 levels, and expressed in arbitrary units (a.u.). Data are expressed as means ± S.E., n = 5 rats. Values not sharing a common letter are significantly different (*p* < 0.05). NFAT5: Nuclear factor of activated T cells 5; AR: Aldose reductase; SDH: Sorbitol dehydrogenase; Smit: Sodium/Myoinositol cotransporter.

In both physiological and pathological cardiac hypertrophy, cardiomyocytes increase in size. Cellular volume homeostasis is maintained by activating specific membrane transport and/or metabolic processes that are essential for normal cell function and survival [34]. The nuclear factor of activated T cells 5 (NFAT5, also named TonEBP) is a transcription factor that regulates these metabolic and transport mechanisms to control cell volume in its normal state. However, as shown in Figure 5A, the mRNA levels of this transcription factor did not differ between the three experimental groups. Curiously, the gene expression of aldose reductase (AR), an enzyme involved in the conversion of glucose into sorbitol and that is regulated by NFAT5, was significantly higher in the offspring of fructose-fed mothers in comparison to the control group. This increase occurred regardless of the diet these descendants consumed during their own pregnancy (Figure 5B). Given that the mRNA gene expression of sorbitol dehydrogenase (SDH), the other polyol pathway enzyme that converts sorbitol into fructose, was not affected (Figure 5C), we can assume that cardiomyocytes in the progeny from fructose-fed mothers would be clearly accumulating sorbitol, an organic osmolyte [35]. To confirm this accretion of sorbitol, we measured the gene expression of the Sodium/Myoinositol cotransporter (Smit). As shown in Figure 5D, Smit was significantly downregulated in FC and FF dams compared to that in the CC group. These opposite profiles for AR and Smit have also been reported in previous studies [36].

## 4. Discussion

Fructose intake has been demonstrated to induce cardiac hypertrophy. This hypertrophy has been linked to several factors, including insulin resistance, autophagy, mitochondrial oxidative stress and dysfunction, and activated fructolysis, which supports anabolic growth [4,7,25,27]. Furthermore, if fructose consumption occurs during pregnancy, heart hypertrophy has also been described in dams, accompanied by fructose-induced oxidative stress and accordingly, glucose dysregulation and lipid, glycogen, and lactate accumulation [13]. Interestingly, maternal consumption of a solid diet containing 60% of fructose can, through a fetal programming mechanism, alter myocardial gene expression making the offspring prone to cardiac hypertrophy. In fact, these male descendants from fructose-fed dams, after being subjected to a ventricular pressure overload, presented exacerbated cardiac remodeling and hypertrophy compared to descendants from control mothers [14]. On the other hand, we have demonstrated that, in contrast to male offspring, female offspring from fructose-fed mothers did not present any differences compared to descendants from control mothers [37]. However, we discovered that they had a hidden phenotype that was revealed either upon re-exposure to liquid fructose or when they became pregnant. Thus, fructose intake in female progeny from fructose-fed mothers induced exacerbated dyslipidemia and steatosis in comparison to progeny from control dams [38]. In addition, pregnancy, a physiological state that significantly alters metabolic and hormonal responses, was sufficient to produce lower insulin sensitivity, higher leptinemia, and lipid accretion in the liver and placenta of descendants from fructose-fed dams versus progeny from control dams. Moreover, the situation dramatically worsened when both fructose re-exposure and pregnancy occurred simultaneously in progeny from fructose-fed mothers, since they also developed hyperinsulinemia [19].

It is well known that cardiac hypertrophy could lead to a heart attack or heart failure. Therefore, the current report is a follow-up study to determine whether metabolic changes occurring during pregnancy in the progeny of fructose-fed mothers could provoke a hypertrophic heart. Moreover, this situation could worsen after consuming liquid fructose during pregnancy.

Interestingly, pregnant females from fructose-fed mothers exhibited significant cardiac hypertrophy in comparison to pregnant females from control mothers (CC), independent of whether they consumed liquid fructose (FF) or not (FC) during their own pregnancy. This situation was specific to the heart, as no similar changes were observed in the liver or kidney, confirming the organ-specific effects of maternal fructose intake. Previous studies have shown that fructose intake can cause heart hypertrophy in male mice consuming a 60% high fructose solid diet [25] for 10 weeks or drinking 10% liquid fructose for 20 weeks [4]. However, the conditions reported by these authors involved a higher fructose content and/or a longer supplementation than those used in our study. Other authors have also reported heart hypertrophy in pregnant rats consuming a standard chow diet and liquid fructose [13]. However, importantly, heart hypertrophy found in the FC group in the present study was induced by maternal fructose intake and not by direct fructose consumption. In addition, hypertrophy caused by maternal fructose (observed in the FC group) was further exacerbated in the FF group by the direct intake of fructose. This was confirmed by the measurement of the DNA content as an index of cellularity. Thus, although there was no significant difference in the HW/BW ratio between the FC and FF groups, the cardiac DNA content was diminished in the FC dams and drastically reduced in the FF group. Other authors have described remodeling and changes in cardiomyocytes induced by maternal fructose intake in male rat descendants from mothers consuming 60% fructose in a solid diet during pregnancy [14]. Curiously, in Leu´s study, heart weight in male descendants from fructose-fed mothers was unchanged compared to that in males from control dams. However, after being subjected to ventricular pressure overload, the consequent heart hypertrophy was found to be exacerbated by maternal fructose intake. A similar situation could possibly occur in our study, since pregnancy is a situation in which physiological heart hypertrophy appears [6], but here, the effects are exacerbated by maternal fructose feeding. In accordance with this, an augmented expression of fetal genes, such as NPPB or βMhc, is commonly observed in pathological hypertrophy, but it is either unchanged or decreased in physiological hypertrophy [6]. In our study, the offspring from fructose-fed mothers, regardless of the diet consumed during their own pregnancy, tended to have increased NPPB gene expression both in FC and FF dams versus control pregnant rats (CC), suggesting pathological hypertrophy.

It has been reported that pathological hypertrophy can be induced, among other causes, by storage defects of lipids, glycogen, and misfolded proteins [6]. In fact, gestational fructose exposure has been demonstrated to increase cardiac triglyceride, glycogen, lactate, and uric acid contents [13]. According to these authors, an increase in PDK4 is the cause of these storage defects. Interestingly, all of these effects were attenuated by glutamine treatment, a recognized agent that can mitigate metabolic cardiac stress. Nevertheless, no storage problems or changes in PDK4 gene expression were observed in our study. Furthermore, although we did not use any glutamine treatment, glutaminase gene expression, an enzyme responsible for glutamine catabolism, was augmented in FF dams, suggesting lower protection from this amino acid against gestational fructose exposure-induced cardiac hypertrophy. In addition, Olaniyi and Olatunji (2019) [13] observed oxidative stress in fructose-fed dams, which was also corrected by glutamine treatment. According to these previous studies, we found that whereas FC dams did not show changes in glutamine metabolism or oxidative stress, both acid uric content and protein carbonyls were augmented in dams that drank liquid fructose (FF) in their pregnancy. Therefore, these effects on glutamine metabolism and oxidative stress, possibly related to cardiac hypertrophy, observed both in pregnant rats subjected to fructose exposure reported by Olaniyi and Olatunji and in FF mothers in the present study, were caused by direct fructose ingestion and not by maternal fructose intake.

Autophagy is a hydrolysis process that degrades intracellular components such as lipids and proteins, and it is important to regenerate nutrients and recycle impaired proteins and organelles [29]. Consequently, defects in autophagy can lead to storage disorders. In addition, it has been demonstrated that cardiac stress during the progression of heart failure promotes cell death and induces individual cardiomyocyte dysfunction through the inhibition of autophagy and mitophagy [6]. Curiously, in a previous report, 60% fructose supplementation in a solid diet for 12 weeks in male mice led to myocardial autophagy activation associated with insulin resistance, NPPB mRNA gene expression diminution, and no evident cardiac hypertrophy signs [27]. In contrast, many other reports have described that high fructose feeding causes hepatic autophagy inhibition, leading to fatty liver and ER stress [28,29]. Since enhanced autophagy can alleviate metabolic and storage disturbances through phagocytosis of liposomes, clearance of damaged cell fragments, and elimination of ER stress, the diminution of autophagy that we observed in FF dams by affecting Ruvbl1 and, mainly, ATG7 gene expression, would promote the hypertrophic situation observed in these rats.

Myocardial hypoxia can also lead to pathological hypertrophy. Moreover, hypertrophy can increase HIF1α expression, whereas hypoxic conditions can stabilize HIF1α and activate its target genes. HIF1α is a transcription factor that controls oxygen homeostasis by regulating angiogenesis, vascular remodeling, and glucose metabolism [6]. In fact, HIF1α activation serves to adapt to acute ischemic events and pressure overload. However, chronic activation of HIF1α can become pathologic, leading to energy deficiency, contractile failings, and cell death [7]. Metabolic reprogramming controlled by HIF1α also coordinates the enhancement of both glucose and fructose uptake and flux [7]. Thus, HIF1α induces glucose uptake as well as major glycolytic genes. Studies in humans and animals have shown that increases in HIF1α expression induced by pressure overload promote fructolysis. This augmented fructolysis produces metabolites that serve as precursors for lipid, nucleic acid, and amino acid biosynthesis, thereby increasing the biosynthetic capacity for tissue expansion and hypertrophic growth [6]. However, in the present study, the expression of glycolytic and fructolytic genes did not appear to be affected in the hearts of FC and FF rats compared to the CC group. Nevertheless, Eberhart et al. (2020) [33] found that classical HIF1α target genes respond clearly after a long period of HIF1α signaling activation, whereas fructolytic genes are only slightly regulated. Interestingly, since the majority of HIF1α target genes measured in the present study were either unchanged or showed a poor response to fructose re-exposure and/or maternal fructose intake, the increase found in VEGFα and BNIP3 gene expression could be produced through other mechanisms, such as epigenetic processes, as previously demonstrated by other authors [39]. In agreement with this, the effects observed in BNIP3 and VEGFα were clearly caused by maternal fructose intake, since direct liquid fructose intake did not produce any additional changes (FF vs. FC dams). The relationship between VEGFα and the heart is controversial. On one hand, it has been reported that VEGFα has beneficial effects since it contributes to well-coordinated cardiomyocyte growth and angiogenesis [6]. On the other hand, VEGFα has been shown to provoke harmful effects because it is produced by cardiomyocytes during inflammation and mechanical stress. Supporting this negative effect, high concentrations of VEGFα have been found in patients with diverse cardiovascular diseases and are correlated with unfavorable prognosis and disease severity [40]. Additionally, it has been described that increased BNIP3 expression in cardiomyocyte diastolic dysfunction produces mitochondrial calcium overload, mitochondrial dysfunction, and a decline in cardiac energetics. Conversely, BNIP3 has also been proposed as a potential therapeutic target for the treatment of diastolic heart failure [41]. Interestingly, mitochondrial function disorders have also been previously found in the pathogenesis of high fructose-induced cardiac hypertrophy [4]. The potential role of BNIP3 and/or VEGFα in cardiac hypertrophy induced directly or indirectly by maternal fructose intake deserves further research.

The cellular basis of cardiac hypertrophy is enlargement of the cardiac myocyte, which is perfectly distinguishable from hyperplasia since the cell number remains, but the cell size increases. Cardiac myocytes can adjust their volume because it is a key property of all mammalian cells, and it is particularly crucial in cardiac cells in the heart, since it is directly linked to cell death [42]. Cells respond to volume perturbations by activating membrane transport systems and/or metabolic processes that result in net solute loss or gain of osmotically active solutes, inorganic ions, or small organic molecules, termed organic osmolytes. The accumulation of organic osmolytes is mediated either by energy-dependent transport from the external environment or by changes in the rates of osmolyte synthesis and degradation [34]. Furthermore, high concentrations of protective organic osmolytes stabilize the protein structure. Therefore, the accretion of these chemical chaperones could be indicative of protein misfolding and ER stress. The main molecules that play this role are amino acids, choline, creatine, sorbitol, inositol, and taurine. NFAT5 is a transcription factor that mediates hypertonicity-induced transcription of enzymes and transporters that control the intracellular content of organic osmolytes. Indeed, hypertonicity can activate the regulation, abundance, and nuclear distribution of NFAT5. In the present study, although the mRNA content of NFAT5 remained unchanged in FF and FC dams, its transcriptional activity was stimulated since AR gene expression was augmented in both FC and FF rats. Interestingly, AR has been demonstrated to be activated during myocardial ischemic injury; moreover, AR inhibitors have been shown to protect rat hearts from ischemia reperfusion injury [35]. AR is the enzyme responsible for the formation of sorbitol from glucose. Additionally, we found that SDH expression was not affected in the present study. Therefore, the increased AR expression observed in FC and FF dams in comparison to CC dams, along with unchanged SDH expression, could lead to an accumulation of sorbitol in these rats. In contrast, Smit gene expression was decreased in FC and FF rats. Although Smit transcription is also regulated by NFAT5, it has been described that sorbitol accumulation can coincide with a diminution in the abundance of Smit mRNA. In fact, several studies have demonstrated that if sorbitol accumulation is blocked using AR inhibitors, the downregulation of the Smit mRNA is prevented. Interestingly, this mechanism might explain the depletion of tissular myo-inositol found during hyperglycemia, which has been related to the pathogenesis of diabetic complications [36].

## 5. Conclusions

In conclusion, we highlight that our findings in the FC group in the present study confirm our previous results [19,38], demonstrating that maternal fructose induces a hidden phenotype through some fetal programming mechanism that can be revealed by pregnancy. Interestingly, pregnancy was sufficient to induce heart hypertrophy in the descendants of fructose-fed mothers. This hypertrophy was mainly induced by the activation of HIF1α and increased osmolality. In addition, liquid fructose intake significantly potentiated the effects of maternal fructose in the progeny of fructose-fed dams, since FF rats not only showed the detrimental changes observed in FC rats but also experienced oxidative stress, lower glutamine protection, and lower autophagy compared to CC dams. Since FF mothers showed more pathways affected than FC dams, this situation possibly explains why they had similar hypertrophy in their hearts (22% and 23% versus CC, for FC and FF, respectively), but a significantly lower DNA content in FF rats (48% vs. CC) than in FC dams (27% vs. CC), proving that the combination of fructose intake and pregnancy in descendants of fructose-fed mothers caused higher heart hypertrophy than pregnancy alone.

## Figures and Tables

**Table 1:** Body and tissue weights of pregnant rats from control mothers (CC) or pregnant rats from fructose-fed mothers subjected (FF) or not (FC) to fructose intake throughout their own pregnancy.

	CC	FC	FF
Body weight (conceptus free) (g)	286.2 ± 3.6 ^B^	261.5 ± 6.9 ^A^	268.9 ± 1.7 ^AB^
Liver weight (g)	12.0 ± 0.5	10.5 ± 0.4	11.3 ± 0.5
Kidney weight (g)	0.68 ± 0.04	0.72 ± 0.03	0.68 ± 0.04
Heart weight (g)	0.91 ± 0.08	1.05 ± 0.03	1.10 ± 0.00

Data are expressed as the mean ± S.E., n = 5 rats. Values not sharing a common letter are significantly different (*p* < 0.05).

## Data Availability

The original contributions presented in the study are included in the article/Supplementary Materials, and further inquiries can be directed to the corresponding author.

## Supplementary Materials

The following supporting information can be downloaded at: https://www.mdpi.com/article/10.3390/foods13182944/s1, Table S1: Hepatic lipids and mRNA gene expression of pregnant rats from control mothers (CC) or pregnant rats from fructose-fed mothers subjected (FF) or not (FC) to fructose intake throughout their own pregnancy.
