# Supplementary material for: Cardiac Hypertrophy in Pregnant Rats, Descendants of Fructose-Fed Mothers, an Effect That Worsens with Fructose Supplementation
Source: Foods. 2024 Sep 18;13(18):2944. doi: 10.3390/foods13182944 (PMC11431301; doi:10.3390/foods13182944)
Supplement: Supplementary file 1 [file foods-13-02944-s001.zip › manuscript.v8-Table S1.pdf]

Table S1. Hepatic lipids and mRNA gene expression of pregnant rats from control mothers (CC) or pregnant rats from fructose-fed mothers subjected (FF) or not (FC) to fructose intake throughout their own pregnancy

	**CC**	**FC**	**FF**
Total Lipids (mg/g of tissue)	69.1 ± 5.3	109.5 ± 30.3	65.1 ± 4.8
Cholesterol (mg/g of tissue)	1.13 ± 0.04	1.03 ± 0.04	1.15 ± 0.03
Phospholipids (mg/g of tissue)	66.8 ± 3.2	58.9 ± 2.2	60.9 ± 3.3
KHK mRNA (a.u.)	0.918 ± 0.05	1.145 ± 0.11	1.063 ± 0.07
AldoB mRNA (a.u.)	1.17 ± 0.34	0.905 ± 0.07	0.88 ± 0.14
LDHA mRNA (a.u.)	0.852 ± 0.07	0.851 ± 0.02	0.95 ± 0.02
PFKB3 mRNA (a.u.)	0.86 ± 0.11	0.96 ± 0.02	0.899 ± 0.18

Data are expressed as means ± S.E., n = 5 rats.
