# Supplementary material for: Cardiac Hypertrophy in Pregnant Rats, Descendants of Fructose-Fed Mothers, an Effect That Worsens with Fructose Supplementation
Source: Foods. 2024 Sep 18;13(18):2944. doi: 10.3390/foods13182944 (PMC11431301; doi:10.3390/foods13182944)
Supplement: Supplementary file 1 [file foods-13-02944-s001.zip › Raw data of Figures.pdf]

		HW/BW Heart	LW/BW Liver	KW/BW Kidney	NPPB	βMhc	Troponin I	µg DNA/mg tissue
**CC**	**2CC2**	0,0027	0,0445	0,0024	0,852	1,453	0,892	0,308
	**2CC3**	0,0037	0,0421	0,0028	1,573	1,051	0,941	0,190
	**2CC4**	0,0029	0,0358	0,0025	0,502	0,882	0,761	0,360
	**2CC5**	0,0039	0,0416	0,0025	0,971	0,826	0,856	0,156
	**2CC6**	0,0032	0,0389	0,0022	1,531	0,898	0,758	0,173
**FC**	**2FC1**	0,0042	0,0401	0,0025	3,213	0,784	0,951	0,133
	**2FC2**	0,0038	0,0355	0,0027	2,046	0,959	0,654	0,260
	**2FC4**	0,0045	0,0440	0,0029	1,895	1,853	0,717	0,124
	**2FC5**	0,0036	0,0405	0,0029	0,831	0,679	0,685	0,182
**FF**	**2FF1**	0,0043	0,0418	0,0024	3,810	0,577	1,214	0,128
	**2FF2**	0,0041	0,0386	0,0026	1,351	1,092	0,853	0,118
	**2FF3**	0,0042	0,0484	0,0030	3,416	1,408	0,663	0,167
	**2FF4**	0,0040	0,0448	0,0022	1,480	0,869	0,633	0,133
	**2FF5**	0,0037	0,0397	0,0026	0,300	0,690	0,808	0,112

	Triglyceride (mg/g prot)	Lactate (mg/g prot)	Uric acid (mg/g prot)	Proteín carbonyls (nmol/mg prot)	Glutamine synthetase	Glutaminase	Glutamate dehydrogenase	PDK4
**2CC2**	381,20	54,07	0,59	1,05	0,77	1,02	1,26	0,98
**2CC3**	41,16	25,86	0,39	2,33	1,43	1,02	1,24	0,58
**2CC4**	33,10	38,27	0,56	1,79	1,27	0,91	0,77	2,68
**2CC5**	89,82	20,81	0,31	0,69	0,91	1,18	0,91	1,04
**2CC6**	37,80	36,71	0,71	2,33	0,79	0,89	0,92	0,63
**2FC1**	486,08	42,20	0,82	5,55	0,85	1,25	1,59	1,67
**2FC2**	308,66	48,36	0,77	0,79	1,18	1,01	1,27	2,46
**2FC4**	61,44	22,33	0,53	2,04	1,23	0,99	1,15	0,81
**2FC5**	119,32	33,00	0,50	2,32	1,26	0,99	1,14	1,29
**2FF1**	91,83	43,27	1,14	4,08	0,86	1,24	1,10	1,43
**2FF2**	177,79	25,09	0,38	6,19	1,89	1,31	1,53	5,17
**2FF3**	49,98	35,69	0,74	3,85	0,96	1,37	1,19	0,57
**2FF4**	187,86	46,53	0,83	3,79	0,93	1,13	1,00	1,58
**2FF5**	33,87	34,33	0,76	2,65	1,06	1,12	1,25	0,64

	MAP1LC3β	Lamp2	Ruvbl1	ATG7
**2CC2**	0,871	0,880	0,957	1,092
**2CC3**	1,326	1,115	1,140	1,055
**2CC4**	0,973	0,953	0,804	0,906
**2CC5**	0,969	0,956	1,068	1,016
**2CC6**	0,918	1,117	1,067	0,942
**2FC1**	0,827	0,737	0,619	0,726
**2FC2**	1,108	0,885	0,894	0,993
**2FC4**	1,150	1,144	0,991	1,073
**2FC5**	0,983	0,823	0,824	0,640
**2FF1**	1,046	0,778	0,848	0,752
**2FF2**	1,539	1,234	1,103	1,050
**2FF3**	1,113	1,003	0,791	0,754
**2FF4**	1,041	1,015	0,768	0,709
**2FF5**	0,950	1,029	0,564	0,642

	HIF1α	MCT1	PDK1	GLUT1	VEGFα	BNIP3
**2CC2**	1,189	1,252	1,399	0,855	1,128	0,733
**2CC3**	1,240	1,418	0,974	1,361	1,106	1,083
**2CC4**	0,810	0,652	0,669	0,937	1,098	0,794
**2CC5**	0,922	1,077	1,060	1,043	0,908	0,799
**2CC6**	0,908	0,802	1,035	0,880	0,804	0,823
**2FC1**	1,463	1,337	1,635	1,141	1,088	1,242
**2FC2**	1,034	1,208	1,349	0,949	1,316	1,121
**2FC4**	1,809	1,660	1,176	1,731	1,489	1,132
**2FC5**	1,230	1,145	1,063	0,962	1,361	1,280
**2FF1**	1,175	1,307	1,401	1,161	1,130	1,174
**2FF2**	1,744	1,558	1,861	2,062	2,379	1,251
**2FF3**	1,674	1,226	1,527	1,168	1,441	0,975
**2FF4**	1,007	1,144	1,094	1,107	1,361	0,846
**2FF5**	1,571	1,335	1,241	1,611	1,083	1,336

	NFAT5	AR	SDH	Smit
**2CC2**	1,430	1,138	1,103	1,120
**2CC3**	0,996	1,438	1,327	0,865
**2CC4**	1,218	0,700	0,700	0,941
**2CC5**	0,718	1,032	1,044	0,793
**2CC6**	0,803	0,846	0,935	0,573
**2FC1**	0,825	1,689	1,202	0,803
**2FC2**	1,141	1,284	1,036	0,636
**2FC4**	1,522	1,475	1,388	0,811
**2FC5**	0,782	1,402	1,296	0,536
**2FF1**	0,552	1,324	1,024	0,674
**2FF2**	1,213	1,801	1,483	0,654
**2FF3**	1,119	1,658	1,268	0,546
**2FF4**	0,926	1,510	0,994	0,635
**2FF5**	0,745	1,527	1,241	0,510
