# Supplementary material for: Cardiac Hypertrophy in Pregnant Rats, Descendants of Fructose-Fed Mothers, an Effect That Worsens with Fructose Supplementation
Source: Foods. 2024 Sep 18;13(18):2944. doi: 10.3390/foods13182944 (PMC11431301; doi:10.3390/foods13182944)
Supplement: Supplementary file 1 [file foods-13-02944-s001.zip › Raw data of Table 1.pdf]

	BW (conceptus free) (g)	LW (g)	KW (g)	HW (g)
**2CC2**	294,123	13,1	0,7	0,8
**2CC3**	289,54	12,2	0,805	1,07
**2CC4**	237,32	8,5	0,6	0,7
**2CC5**	283,65	11,8	0,7	1,1
**2CC6**	277,36	10,8	0,6	0,9
**2FC1**	264,41	10,6	0,661	1,1
**2FC2**	262,32	9,3	0,7	1
**2FC4**	243	10,7	0,7	1,1
**2FC5**	276,34	11,2	0,8	1
**2FF1**	253,45	10,6	0,6	1,1
**2FF2**	269,44	10,4	0,7	1,1
**2FF3**	264,34	12,8	0,8	1,1
**2FF4**	272,31	12,2	0,6	1,1
**2FF5**	269,38	10,7	0,7	1
